# Minority Drug-Resistant HIV-1 Variants in Treatment Naïve East-African and Caucasian Patients Detected by Allele-Specific Real-Time PCR

**DOI:** 10.1371/journal.pone.0111042

**Published:** 2014-10-21

**Authors:** Halime Ekici, Wondwossen Amogne, Getachew Aderaye, Lars Lindquist, Anders Sönnerborg, Samir Abdurahman

**Affiliations:** 1 Department of Laboratory Medicine, Division of Clinical Microbiology, Karolinska Institutet, Stockholm, Sweden; 2 Department of Medicine, Faculty of Medicine, Addis Ababa University, Addis Ababa, Ethiopia; 3 Department of Medicine, Unit of Infectious Diseases, Karolinska Institutet, Karolinska University Hospital Huddinge, Stockholm, Sweden; 4 Department of Science and Technology, Örebro Life Science Center, Örebro University, Örebro, Sweden; University of Athens, Medical School, Greece

## Abstract

**Objective:**

To assess the presence of two major non-nucleoside reverse transcriptase inhibitors (NNRTI) drug resistance mutations (DRMs), Y181C and K103N, in minor viral quasispecies of treatment naïve HIV-1 infected East-African and Swedish patients by allele-specific polymerase chain reaction (AS-PCR).

**Methods:**

Treatment naïve adults (n = 191) with three epidemiological backgrounds were included: 92 Ethiopians living in Ethiopia; 55 East-Africans who had migrated to Sweden; and 44 Caucasians living in Sweden. The *pol* gene was analysed by standard population sequencing and by AS-PCR for the detection of Y181C and K103N.

**Results:**

The Y181C was detected in the minority quasispecies of six Ethiopians (6.5%), in two Caucasians (4.5%), and in one East-African (1.8%). The K103N was detected in one East- African (1.8%), by both methods. The proportion of mutants ranged from 0.25% to 17.5%. Additional DRMs were found in all three treatment naïve patient groups by population sequencing.

**Conclusions:**

Major NNRTI mutations can be found by AS-PCR in minor quasispecies of treatment naïve HIV-1 infected Ethiopians living in Ethiopia, in East-African and Caucasian patients living in Sweden in whom population sequencing reveal wild-type virus only. Surveys with standard sequencing are likely to underestimate transmitted drug resistance and the presence of resistant minor quasispecies in treatment naïve patients should be topic for future large scale studies.

## Introduction

At the end of 2012, about 9.7 million people were given antiretroviral therapy (ART) in low- and middle-income countries (LMIC) [1]. In Ethiopia, the number of patients on ART increased from 11 000 in 2004 to 222 700 in 2010 [2]. This widespread use of ART increases the risk for transmission of drug-resistant HIV-1 (TDR) [3]–[8]. Indeed, a steady increase of TDR in treatment naïve patients has been reported in sub-Saharan Africa [8]–[12]. In Europe, migration of HIV infected individuals from LMIC contributes substantially to the epidemic.

The standard genotyping assay detects mutations that are present in >20% of the viral population [13]–[15]. Recently, more sensitive techniques have been developed, such as allele-specific PCR (AS-PCR) [16]–[18] and ultra-deep sequencing [19], [20]. These methods have been used so far only to a limited extent in clinical care, despite that such minor quasispecies may influence the outcome of ART [3]–[5]. However, no study has yet, to our knowledge, reported the presence of drug-resistant minor HIV-1 variants in treatment naïve patients living in LMIC with the exception of women who have received prophylaxis against mother-to-child transmission. In addition, there are only two studies describing TDR in Ethiopians [21], [22] using standard genotypic assays. In Sweden, the prevalence of TDR has been reported to be low during 2003–2010 [23], although no studies using sensitive assays have been conducted.

The aim of our study was therefore to analyse the presence of NNRTI-resistant minor HIV-1 variants in three treatment naïve patient-populations with different epidemiological background; Ethiopians living in Ethiopia, East-Africans who had migrated to Sweden, and in Caucasian patients living in Sweden. For this purpose, the most prominent NNRTI mutations, K103N and Y181C, were investigated by a highly sensitive AS-PCR and the results were compared with direct population sequencing.

## Materials and Methods

### Clinical samples

Plasma samples, which had been stored at −80°C until used, were collected from treatment naïve East-Africans (n = 55) and Caucasians (n = 44), who were followed during 2002–2013 at the Infectious Disease Clinic, Karolinska University Hospital, Stockholm, Sweden, and from Ethiopians living in Addis Ababa, Ethiopia (n = 92) (Table 1). Females who had been given prophylaxis against mother-to-child transmission were not included. The selection of the patients in Sweden was done randomly among those where a baseline sample obtained at diagnosis was available. The East-Africans had migrated from the following countries: Ethiopia (n = 26), Eritrea (n = 23), Somalia (n = 2), Zimbabwe (n = 2), Tanzania (n = 1), Kenya (n = 1). The Ethiopians lived in the capital Addis Ababa and were recruited in 2008–2009 as part of a clinical research cohort [24] before ART (stavudine or zidovudine/3TC/nevirapine) was initiated.

**Table 1:** Characteristics of treatment naïve HIV-1 infected patients included in the study.

Characteristics	Ethiopians in Ethiopia	East Africans in Sweden	Caucasians in Sweden
Number	92	55	44
Sex [n] (%)			
Female	66 (72)	29 (52)	8 (19)
Male	26 (28)	26 (48)	36 (81)
Age [years] (median, IQR^a^)	35 (29–41)	36 (30–41)	41 (38–52)
CD4 count [cells/µl] (median, IQR)	100 (56–144)	204 (122–317)	336 (210–504)
HIV load [log_10_ copies/ml (median, IQR)]	5.4 (5–5.8)	4.9 (4.1–5.6)	5.0 (4.1–5.8)
HIV subtype [n] (%)	C (100)	B (2), C (98)	B (98), C (2)
Transmission			
Heterosexual [n] (%)	92 (100)	53 (96)	8 (18)
Homosexual	-	-	25 (57)
IVDU^b^	-	-	11 (25)
Unknown	-	2 (4)	-
Patients with DRM^c^	6	2	2

a IQR, interquartile range;

b IVDU, intravenous drug use;

c DRM, drug resistant mutation.

### Ethics statement

Ethical approvals were obtained from the Regional Ethics Committee, Stockholm (Dnr: 2006/1367-31/4), the Ethiopian Science and Technology Agency (Ref. No. RPHE/126-83/08) and from the Drug Administration and Control Authority of Ethiopia (Ref. No. 02/6/22/17). All participants gave written informed consent.

### Construction of K103N & Y181C plasmid controls

Plasmid DNA standards were generated as described [18]. Briefly, a 1203 bp DNA fragment of the RT gene of HIV-1NL4-3 strain was amplified using primer pairs B1 and B2 (Table S1). The resulting fragment was gel purified and cloned into pCR4-TOPO cloning vector (Invitrogen). The resulting clone, pCR4-RT, was then used as a template for QuickChange II Site-directed PCR mutagenesis (Stratagene) to create the K103 and Y181 mutant plasmids. The three desired single-mutant plasmids used as standards, K103N-AAC, K103N-AAT, and Y181C-TGT were created using mutagenic primer sequences containing the following substitutions: K103N (AAA→AAC, AAA→AAT) and Y181C (TAT→TGT). All plasmids DNAs were propagated in Escherichia coli XL10-Gold and purified by using a Miniprep Purification kit (Qiagen) and the identity of each sequence was confirmed by sequencing (MWG Operon, Germany).

### Allele-specific real-time PCR primer design

**Figure 1:**
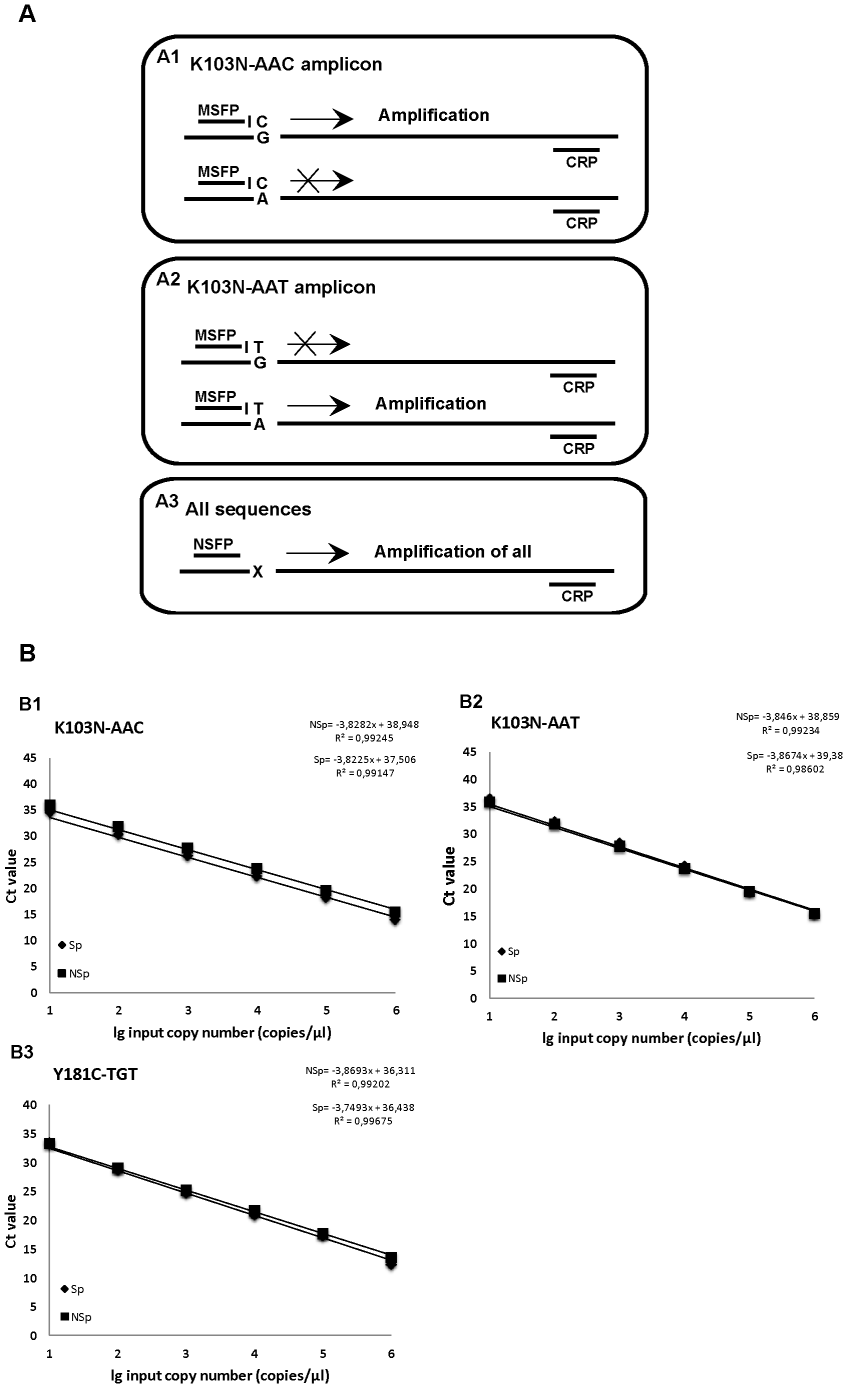
Schematic overview of allele-specific real-time PCR primers design and setting the standard curve analysis. In presence of K103N-AAC (A1) and K103-AAT (A2) mutants, only the specific mutant amplicons will be amplified when using mutant specific forward (MSFP) and a common reverse primer (CRP), respectively. The total population of sequences in the reaction are amplified using non-specific forward primer (NSFP) and the CRP (A3). An intentional mismatch at the penultimate base (indicated with I) was introduced in the allele-specific real-time PCR primers in order to increase the specificity and minimize the risk for amplification of the wild type allele. Mutant specific (Sp) and non-specific (NSp) standard curves of K103N AAC allele (B1), K103N AAT allele (B2) and Y181C TGT allele (B3) are in parallel with each experiment. The copy number of each mutant specific and total population of sequences amplifications of clinical samples were determined using such standard curves that has been run in duplicate, parallel with each sample. The quantity of the patients' mutant specific and the total population of sequences (amplified with non-specific primer) was then determined by comparing the samples Ct value with those of the specific and non-specific standard curves derived from the standard plasmid controls using the corresponding primers. The percentage of mutant specific sequences was then determined by dividing the quantity of mutant specific sequence by the quantity of the total sequences and multiplying by 100. Positive samples were repeated at least twice. Correlation coefficients (r^2^) were higher than 0.99. Sp: mutant specific amplification. MSFP, mutant-specific forward primer; CRP, common reverse primer; NSFP, non-specific forward primer. NSp: non-specific amplification (amplify the total population of sequences).

Mutant specific primers refer to primers for the K103N-AAC, K103N-AAT or Y181C-TGT alleles that specifically amplify the mutant sequences. Non-specific primers on the other hand amplify the total population of the sequences in each sample, regardless of the genotype at the specific allele (Figure 1A). The non-specific primers and mutant specific primers for K103N and Y181C mutants were designed by choosing the maximum consensus sequence from alignments of 598 HIV-1 subtype B and C sequences available from the Los Alamos National Laboratories HIV sequence database (www.hiv.lanl.gov). Viral RNAs were reverse transcribed to cDNAs using random hexamers (Invitrogen). The primer binding sites for the subsequent nested-PCR and the AS-PCR assays were 100% complementary to the primer binding sites of all the standards. For amplification of K103 mutants, the same reverse primer (D2, Table S1) was used for all reactions and the 3′-end of the forward primers contained the mutagenic sequences that could specifically amplify the desired mutant. For the Y181 amplification, the reverse primers contained the mutagenic sequence that amplified the desired mutant and the same forward primer (E1, Table S1) was used for all reactions. For total population of HIV-1 quantification, the forward primer for K103 and the reverse primer for Y181 were designed so that the 3′ end of the primer was set just upstream of codon 103 and downstream of codon 181, respectively. In order to increase the selectivity of the assay and minimize amplification of the wild type allele, each allele-specific primer was anchored (−1) at the 3′end of mutant base (depicted as bold and italic “I” in Table S1).

### Standard curves

Using the above wild type and mutant plasmid DNA clones, independent standard curves were generated for each AS-PCR run by making a series of 10-fold dilutions over a range of seven logs. Each sample was analysed in duplicate with mutant specific and non-specific primers of each mutation in parallel, together with no template and HIV-1 positive control. The quantity of unknowns was then determined by comparing the samples Ct value with those standard curves derived from plasmid standard controls using the corresponding primers.

### HIV-1 RNA extraction, reverse transcription and nested PCR

Nucleic acid was isolated using the QIAamp viral RNA extraction mini-kit (Qiagen) from 140 to 1000 µl aliquots of plasma. If the viral load was <10000 copies/ml, virus particles were pelleted by ultracentrifugation at 23 000×g for 1 h at 4°C. Equal amounts of extracted RNAs were reverse transcribed to cDNA. The cDNA synthesis was performed using the Superscript III First Strand Synthesis SuperMix (Invitrogen) with an initial incubation for 10 min at 25°C followed by 50 min incubation at 50°C and terminated by a final deactivation of the enzyme at 85°C for 5 min. The first round PCR was performed using the primer pairs A1 and A2, followed by a second-round PCR using the B1 and B2 primer pairs (Table S1). The resulting nested-PCR product of 1203 bp DNA fragment was analysed on agarose gel and extracted using the Qiagen's gel extraction kit. The concentration of gel purified DNAs were determined and equal amounts of PCR product corresponding to 10^6^ copies was then used for AS-PCR. The nested PCR was performed using the Expand High Fidelity Plus PCR System (Roche Diagnostics).

### Detection and quantification of K103N and Y181C mutants by AS-PCR

The AS-PCR was performed on the ABI sequence Detector System 7500 (Applied Biosystems). Amplification was carried out in a total volume of 25 µl containing 12.5 µl Power SYBR Green PCR Master Mix (Applied Biosystem), 1 µl of forward and reverse primers (10 µM) and 5 µl of template. The PCR cycle conditions were similar for all reactions with the initial denaturation step at 95°C for 10 min (1 cycle), followed by 50 cycles amplification at 95°C for 15 sec and 58°C for 1 min. To confirm amplification specificity, the PCR products were subjected to a final step of melting curve analysis at 85°C for 15 sec. The quantity of each patients' mutant specific or the total population of sequences (amplified with non-specific primer) was determined by comparing the samples Ct value with those of the specific and non-specific standard curves derived from plasmid standard controls using the corresponding primers. Dividing the quantity of mutant sequence by the quantity of total sequences and multiplying by 100 determined the percentage of mutant specific sequences. Positive samples were repeated at least twice.

### Assay validation

The selectivity and accuracy of the assay was evaluated by mixing various proportions of mutant and wild type DNAs. The mixtures of templates in which the proportion of mutant DNAs ranged from 0.01 to 100% were then analysed with each mutant specific (AAC, AAT, or TGT) and non-specific primer pairs that amplified the total populations and confirmed by melting curve analysis. The total amount of input plasmid DNA standards used in all mixtures was the same (10^5^ copies/ml). Establishment of cut-off for the assay background was determined as a mean Ct value from eight independent runs of 100% wild type template with mutant specific primers plus three standard deviations.

### Direct sequencing

Genotypic analysis of the *pol* gene was performed by direct sequencing using nested PCR products (see above) that were purified using the QIAQuick PCR purification kit, before being sent for sequencing to MWG operon (MWG operon, Germany). The two overlapping sequences using B primer pairs (Table S1) produced a fragment of an approximately 1203-bp in the 5′ half of the pol gene. Sequences were edited using the BioEdit software version 7.2.5 (http://www.mbio.ncsu.edu/bioedit/bioedit.html). TDR was identified using the WHO 2009 list of mutations for surveillance of TDR [25] as implemented in the Calibrated Population Resistance tool (v5.0 beta) [26] available at the Stanford HIV Drug Resistance Database (hivdb.stanford.edu).

## Results

### Primers, standard curves and the amplification efficiency

When the specificity and cross-reactivity of each mutant specific primer was tested in a series of experiments using wild type plasmids as a template, there was no cross-reactivity as determined by the differences in Ct values between the wild type and mutant plasmid DNAs. Eight independent measurements of wild type plasmid template with mutant specific primers established the assay background to be 0.024% (SD, 0.018). Amplification of the mutant and wild type plasmid controls with their corresponding mutant specific and non-specific primers, respectively, resulted in a Ct value that was linearly correlated with the amount of input DNA down to 10 copies (Figure 1B). The amplification efficiency of each mutant specific and non-specific sequence was comparable with a correlation coefficient (r^2^) higher than 0.99.

### Sensitivity, specificity and selectivity of the AS-PCR

AS-PCR using a mixture of each mutant and wild type plasmid controls set the sensitivity and discrimination ability of the assay. When equal amounts of mutant (AAC, AAT, or TGT) and wild type plasmids (10^5^ copies) were amplified separately with each of the corresponding mutant specific primer, the differences in Ct (ΔCt) values between the wild type and mutant sequences were either undetectable (no fluorescent signal above the threshold cycle was detected) or were more than twenty.

**Figure 2:**
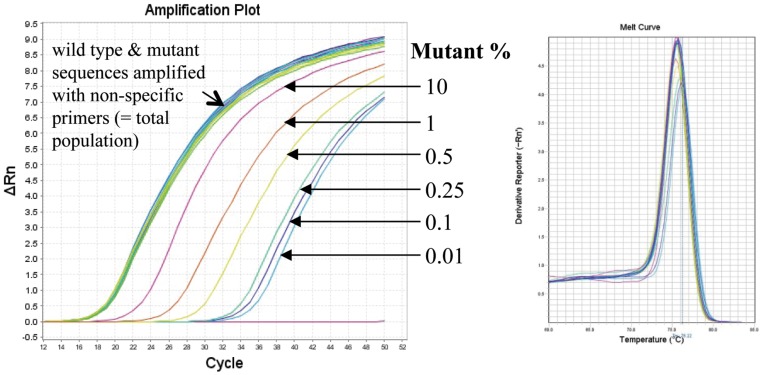
Example of allele-specific real-time PCR amplification curves of cloned wild type and mutant TGT plasmid DNAs at different frequencies (raw data). The specificity and accuracy of the assay was determined by running allele-specific real-time PCR of mixtures of mutant and wild type DNA standards ranging from 0.01 to 100% and amplified with the mutant specific primers in the background of wild type sequence. Amplification of the total population results always in the same Ct values, regardless of the amount of mutant DNAs present in the reaction. WT, wild type.

The specificity and selectivity was tested by mixing mutant and wild type plasmid DNA controls in different proportions (Figure 2). When a mixture of mutant sequence (AAC, AAT, or TGT) ranging from 0.01% to 100% was amplified with the mutant specific primers in the background of wild type sequence, mutant sequences could be detected down to 0.01%. A representative amplification plot for the TGT mutant in the background of the wild type plasmid is shown in Figure 2. The specificity of the AS-PCR amplification was further confirmed by subjecting the PCR products to a final step of melting curve analysis. From replicates of two independent experiments, a conservative accuracy of mutant HIV-1 variants was found to be 0.1% for K103N (AAC and AAT), and 0.25% for Y181C (TGT).

### Reproducibility

Mixtures of a given amount of mutant and wild type sequences were used to determine the intra-assay and inter-assay variability of the AS-PCR. The intra-assay variability was calculated from two independent experiments of quadruplets of mutant and wild type sequence mixtures. The intra-assay coefficient of variation (CVs) ranged from 0.01 to 0.12 for the nominal mutant proportion of 100% and 0.01%, being lower than 40% for the CV for the replicates containing no samples. The inter-assay variability determined from two separate experiments run on different days resulted in CV values ranging from 0.06 to 0.31 for the nominal mutant proportion of 100% and 0.01%, being lower than 42% for the CV for the replicates containing no samples.

### Identification of drug resistance mutations

**Figure 3:**
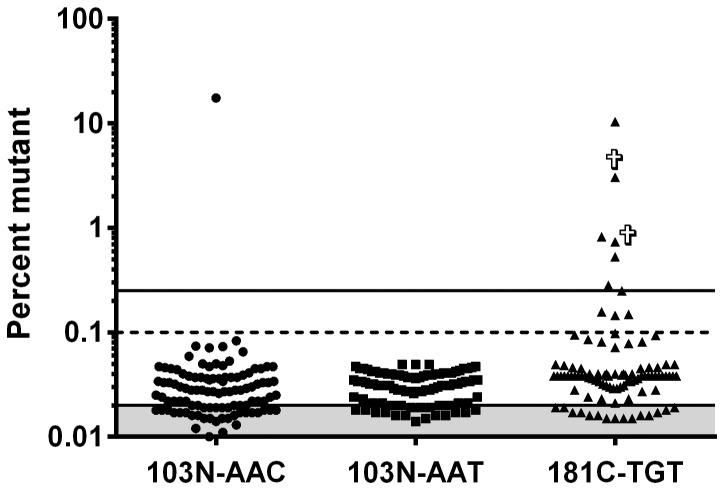
Detection frequency of mutant K103N and Y181C minority variants. Ninety-two treatment naive HIV patients plasma were analysed by allele-specific PCR, detecting the two major NNRTI mutations K103N and Y181C in the reverse transcriptase gene. The limit of detection for the K103 mutations (AAC and AAT) is indicated by a dashed line. The limit of detection for the Y181C mutation (TGT) is indicated by a solid line. Crosses indicate two patients who died within three months. Shaded area indicates the assay background, which was determined as a mean Ct value from eight independent runs of 100% wild type template with mutant specific primers plus three standard deviations.

AS-PCR detected Y181C in five females and one male among the 92 (6.5%) Ethiopian patients from Addis Ababa (Table 2). The proportion of Y181C ranged from 0.25% to 4.5% (Figure 3). In none of these patients, the K103N was detected. Due to the low number of patients with DRM at baseline, it was not possible to study the impact on the outcome of ART. Direct sequencing identified two patients with additional DRM (L100IL, M46L) (Table 2).

**Table 2:** Characteristics of East-African, Caucasian and Ethiopian patients with drug resistance mutations.

							AS-PCR		Direct Sequencing	
ID	Gender	Subtype	Year	Origin	CD4	VL log	Y181C (%)	K103N (%)	RT-region	PI-region
***East Africans living in Sweden***										
**1**	M	C	2003	Ethiopia	190	4.02			M184V	
**2**	M	C	2003	Eritrea	270	5.57			M184V	N88S
**3**	M	C	2005	Eritrea	281	5.15			K101E, Y188L	
**8**	M	C	2009	Eritrea	186	5.13		17.5	K103N	
**10**	F	B	2006	Tanzania	189	5.03	0.8			
***Caucasians living in Sweden***										
**12**	M	B	2011	Belarus	530	2.75	10.3		N.D.^a^	
**13**	M	B	2008	Russia	14	5.22			V106I	
**19**	M	B	2009	Sweden	330	3.57	3.0			
**20**	M	B	2011	Sweden	230	5.29			T215S	
**21**	M	B	2011	Lebanon	960	3.87				L90M
***Ethiopians living in Ethiopia***										
**24**	F	C	2008–2009	Ethiopia	48	6	0.28		L100IL	
**26**	M	C	2008–2009	Ethiopia	184	5.80	4.46		N.D.^a^	
**27**	F	C	2008–2009	Ethiopia	37	5.76	0.85			
**29**	F	C	2008–2009	Ethiopia	115	6	0.53		N.D.^a^	
**43**	M	C	2008–2009	Ethiopia	77	5.50				M46L
**44**	F	C	2008–2009	Ethiopia	90	5.40	0.25			

a ND, not done.

Among the East-Africans who had migrated to Sweden, AS-PCR identified minor NNRTI mutations in two patients (3.6%); one female with Y181C and one male with K103N (Table 2). The proportion of Y181C was 0.8% and K103N 17.5%, respectively. Additional DRM were detected in four patients with direct population sequencing. Of these, two had M184V, two had NNRTI mutations (K101E, Y188L) and one had a PI mutation (N88S).

In the Caucasian patients, AS-PCR detected Y181C in two male patients (4.5%) with a proportion of 3.0% and 10.3%, respectively (Table 2). No K103N was detected. Direct sequencing identified additional TDR in two patients: T215S and L90M, respectively.

## Discussion

Transmission of drug-resistant HIV-1 (TDR) is increasing in Africa as determined by direct population sequencing [12]. However, while the occurrence of drug resistance in minor HIV-1 quasispecies and their impact on treatment outcome are well documented in Europe and North America [3], [4], [6], [20], [27]–[31], no data has to our knowledge been reported from LMIC with the exception of pregnant women who have received nevirapine at delivery. In addition, migrants from LMIC constitute a substantial portion of newly diagnosed patients in Western Europe. Therefore, to gain further knowledge of to which extent direct sequencing underestimates the presence of TDR in African populations, we analysed Ethiopians infected with HIV-1C living in Addis Ababa, and East Africans with HIV-1C living in Sweden and compared the results with that of Caucasians with HIV-1B living in Stockholm, for the presence of the major NNRTI mutations, K103N and Y181C.

Our AS-PCR was shown to be highly sensitive and selective, detecting HIV-1 variants down to 0.01%. Mixing experiments of mutants in the background of wild type showed a conservative accuracy of 0.1% for K103N and 0.25% for Y181C with a high reproducibility. The method is simple and relatively easy to perform, however, careful design of mutant specific primers and appropriate standard curves were needed to minimize the risk for underestimation due to polymorphisms at the primer binding sites in the target sequences. The primers were designed to amplify both HIV-1C and HIV-1B, which are the dominating subtypes world-wide.

The Y181C mutation was found in six of the 92 (6.5%) Ethiopians living in Addis Ababa with mutant frequencies ranging from 0.25% to 4.5%. All patients were newly diagnosed and reported no prior use of ART. Also, none of them had received nevirapine for prophylaxis of mother-to-child transmission. Standard genotypic sequencing identified two additional drug resistance mutations in the RT- and PR-coding region, despite that PI was not used in the Ethiopian health care system at this time point.

DRM were detected also in the treatment naïve East-Africans who had migrated to Sweden as well as in Caucasians living in Stockholm. In both categories of patients, additional NNRTI mutations were identified in the minor quasispecies, although there was a concordance in most patients with the population sequencing. In addition, one PI-related DRM was detected by standard sequencing in both groups. The prevalence of DRM in the minority viral populations found in the East-African and Caucasian group in this study are at the same level as the prevalence of resistance to NNRTIs (3.4%) across Europe, as determined by direct sequencing [32].

The roll-out of ART in sub-Saharan Africa started in 1998 [12]. However, with the widespread use of ART and the lack of adequate virological monitoring, the inevitable consequence is accumulation of DRM. The increase is mostly driven by NNRTI mutations, which could impair the efficacy of available first treatments. Surveys of TDR are based on direct sequencing (WHO) and despite an increasing rate of transmitted and acquired NNRTI resistance, efavirenz or nevirapine are still key components in the first line ART. In case-control studies, minority NNRTI-resistant strains can have an impact on ART [3], [27], [29]. In a recent study from the Swiss HIV cohort [33], the NNRTI-containing, first-line ART was effective in 10 patients with preexisting minority NNRTI-resistant HIV-1 variant. The relatively low prevalence of Y181C in our study (6.5%) is at the same level as in the Swiss study and no major impact on the therapy outcome would be expected if our patients had been given NNRTI-containing therapy The prevalence of drug-resistant minor HIV-1 variants in Ethiopia has not been previously investigated. In fact, only few published data are available on prevalence of primary DRM in Ethiopia [21], [22], in which the frequency have ranged from 0% in Addis Ababa to 2.2% in Gonder, northwest of the country. Using AS-PCR, we found a higher-abundance of drug-resistant minor HIV-1 variants. Thus, transmission of NNRTI-resistant strains seems to occur in Ethiopia, not always detected with standard population sequencing, although the prevalence and the proportion of resistant strains still remained low during the study period of 2008–2009.

In conclusion, major NNRTI mutations can be found by AS-PCR in minor quasispecies of treatment naïve HIV-1 infected Ethiopians living in Ethiopia, in East-African and Caucasian patients living in Sweden. Surveys with standard sequencing may underestimate transmitted drug resistance. AS-PCR or, as we recently described [34], high throughput next generation sequencing with multiplexed amplicon could possibly be more cost-efficient approaches for large scale surveillance of primary DRM in low-middle income countries where routine pre-ART genotypic resistance testing is not standard of care.

## Supporting Information

Table S1 **List of primer sequences used for cloning plasmid DNA used to generate various standard curves for AS-PCR.**
(DOCX)
