# Supplementary material for: Minority Drug-Resistant HIV-1 Variants in Treatment Naïve East-African and Caucasian Patients Detected by Allele-Specific Real-Time PCR
Source: PLoS One. 2014 Oct 21;9(10):e111042. doi: 10.1371/journal.pone.0111042 (PMC4205091; doi:10.1371/journal.pone.0111042)
Supplement: Table S1 — List of primer sequences used for cloning plasmid DNA used to generate various standard curves for AS-PCR. (DOCX) [file pone.0111042.s001.docx]

Supporting Table S1. List of primer sequences and their binding sites.

| Application | Denomination | Sequences (5´→3´) | Nucleotides^*^ |
| --- | --- | --- | --- |
| Nested-PCR | A1 | GAAAGAYTGTACYGAGAGACAGGCTAAT | 2058-2081 |
|  | A2 | TTAATCCCTGGGTAAATCTGACTTG | 3350-3373 |
|  | B1 | AGCAGGAGCYGAARGACAGGG | 2135-2158 |
|  | B2 | TYCCCACTAACTTYTGTATRTCATTG | 3315-3338 |
| K103N-ASPCR | D1-AAC | CMGCAGGGTTAAAAAAGA***I*C** | 2839-2858 |
|  | D1-AAT | CMGCAGGGTTAAAAAAGA***I*T** | 2839-2858 |
|  | D1-ALL | CAYCCMGCAGGGTTAAAAAAG | 2835-2856 |
|  | D2 | CTGTGGRAGSACATTRTAYTG | 2982-3002 |
| Y181C-ASPCR | E1 | TCARTAYAATGTSCTYCCACAGG | 2981-3003 |
|  | E2-TGT | ATACAARTCATCCATRTATTG***I*C** | 3091-3113 |
|  | E2-ALL | CTACATACAARTCATCCATRTATT | 3094-3117 |

* Sequence numbering is relative to the HXB2 laboratory reference strain. In allele-specific real-time PCR (AS-PCR) primers, the mutageneic nucleotide is shown in boldface and underlined. The intentional mismatch (A→I in K103 and T→I in Y181) that has been incorporated at the -1 position of the 3’-end is shown in boldface and italic. This mismatch is incorporated only in the mutant specific primer in order to increase primer specificity.
